# Carnosic acid increases sorafenib-induced inhibition of ERK1/2 and STAT3 signaling which contributes to reduced cell proliferation and survival of hepatocellular carcinoma cells

**DOI:** 10.18632/oncotarget.27687

**Published:** 2020-08-18

**Authors:** Xuening Wang, Priya Gupta, Yasmeen Jramne, Michael Danilenko, Dongfang Liu, George P. Studzinski

**Affiliations:** ^1^Department of Pathology, Immunology and Laboratory Medicine, Rutgers New Jersey Medical School, Newark, NJ 07103, USA; ^2^Department of Clinical Biochemistry and Pharmacology, Faculty of Health Sciences, Ben-Gurion University of the Negev, Beer-Sheva 84105, Israel; ^3^Center for Immunity and Inflammation, New Jersey Medical School, Rutgers the State University of New Jersey, Newark, NJ 07103, USA

**Keywords:** sorafenib, carnosic acid, ERK1/2, STAT3, hepatoma

## Abstract

Hepatocellular carcinoma (HCC) has increasing worldwide incidence but when unresectable lacks curative options. Treatment with a kinase inhibitor Sorafenib (Sf), while initially effective, results in only short increases in patient survival, thus there is a need for improved treatment regimens. Numerous treatment regimens have been explored wherein Sf is combined with other agents, such as non-toxic botanicals like Curcumin or Silibinin. Recently, we have shown that carnosic acid (CA), a component of the food preservative Rosemary Extract, can markedly enhance the cytotoxic actions of Sf in several cell lines derived from HCC, but not in the cell line Hu1545 derived from normal hepatocytes. CA has been shown to enhance Sf-induced cell death in the neoplastic cell lines, principally due to the composite of increased apoptosis and cytotoxic autophagy. In the present study we focused on the mechanisms that underlie the reduced proliferation and survival of HCC cells when CA is added to Sf and how this relates to the increase in Sf-induced DNA damage as well as to the elevation of cytoplasmic levels of reactive oxygen species (ROS). Importantly, the elevation of ROS levels induced by Sf was increased by adding CA. We found that CA enhanced Sf-induced prolongation of cell cycle, and the overall decrease in cell growth was associated with reduced activation of both STAT3 transcription factor (TF) and extracellular signal-regulated protein kinase (Erk)1/2. Our data suggest that a regimen incorporating CA, an inexpensive and non-toxic food additive, in the treatment of advanced HCC merits clinical evaluation.

## INTRODUCTION

Hepatocellular carcinoma (HCC) has worldwide incidence but the advanced cases have few effective curative options. Attempts to improve its prognosis have included the introduction of several multikinase inhibitors into clinical practice for therapy of advanced HCC, but until recently, sorafenib (Sf) was one of the most often used FDA approved systemic drugs for its treatment [1]. In the last few years, a number of other tyrosine kinase inhibitors (TKIs) have been investigated, and lenvatinib (an inhibitor against the VEGFR1, VEGFR2, and VEGFR3 kinases) and cabozantinib (an inhibitor of the tyrosine kinases c-Met, VEGFR2, AXL, and RET) are now also approved as treatments for patients with metastatic HCC [2, 3]. Also, improved patient outcomes were reported in randomized Phase III trials with regorafenib [4] and ramucirumab [5] as second line treatment after Sf alone failed to halt disease progression. Both clinical practice and laboratory studies have mainly focused on Sf, originally developed as a Raf kinase inhibitor known as BAY 43-9006, and marketed as Nexavar (eg [6]).

As with other TKIs, therapy with Sf results in only short increases (about 12 weeks) in patient survival along with considerable toxicity, thus there is a need for improvements of these treatment regimens. The main strategy has been to combine Sf with other cytotoxic agents (eg [7–9]). Combinations of Sf with non-toxic botanicals such as curcumin and silibinin have also been reported [10, 11]. More recently, we have shown that carnosic acid (CA), a polyphenolic antioxidant from the food preservative Rosemary Extract [12], used alone, or together with a vitamin D2 analog, can markedly enhance the cytotoxic actions of a low concentration of Sf in two cell lines derived from HCC, Huh7 and HepG2, but not in the Hu1545 cell line derived from normal hepatocytes. The enhanced cell death in the neoplastic cell lines was shown to be principally due to increased apoptosis and cytotoxic autophagy [13].

In the present study of Sf-treated HCC cells we focused on the mechanisms that underlie the reduced cell proliferation when CA is added to Sf and how this relates to the Sf-induced ROS generation, DNA damage, cell proliferation, and cell survival. We found that CA further increases DNA damage and the cell cycle block initiated by Sf, which correlates with ROS generation, reduced cell proliferation and cell survival, as well as the activation of the transcription factor (TF) STAT3, and of the proliferation-regulatory kinase ERK1/2.

## RESULTS

### Carnosic acid potentiates the prooxidant effect of Sorafenib on HCC cells

**Figure 1:**
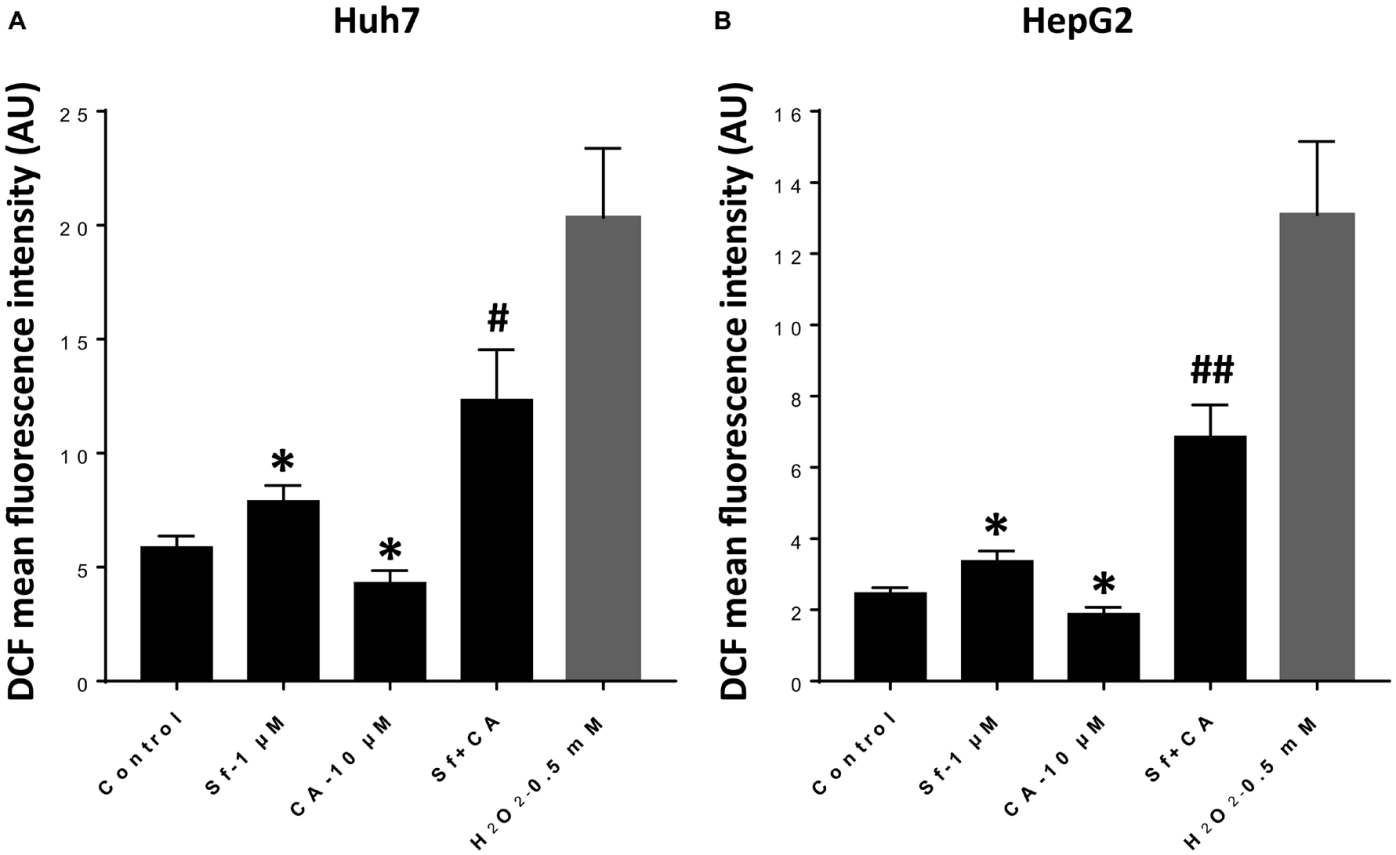
Carnosic acid potentiates sorafenib-induced elevation of cytosolic ROS levels in HCC cells. (**A** and **B**) Mean fluorescence intensity of the DCFH-DA oxidized product dichlorofluorescein (DCF) was measured in Huh7 (A) and HerpG2 (B) cells following treatment with the indicated agents for 24 h. Cells exposed to H_2_O_2_ for 30 minutes were used as the positive control. The data are the means ± SD (*n* = 7 for Huh7; *n* = 6 for HepG2). ^*^ *p* < 0.05 *vs.* control; ^#^ *p* < 0.05; and ^##^ *p* < 0.01 *vs.* Sf alone.

Sorafenib action on HCC cells is known to generate ROS [14, 15], but it has not been determined if the enhancement of the therapeutic effect of Sf by CA [13] is associated with an increased ROS production. We therefore measured cytosolic ROS levels following incubation of Huh7 and HepG2 cells with Sf (1 μM), CA (10 μM), and their combination [13] for 24 h using the fluorescent indicator DCFH [16–20]. Cells treated with the prooxidant H_2_O_2_ (0.5 mM) for 30 min were used as the positive control (Figure 1A). As expected, treatment with Sf alone resulted in the elevation of ROS levels in both HCC cell lines, whereas exposure to the antioxidant CA alone had an opposite effect (Figure 1A and 1B). However, when combined with Sf, CA not only failed to inhibit the prooxidant effect of Sf, but rather, it even further increased ROS generation (Figure 1A and 1B).

### Accentuation of Sf-induced DNA damage by carnosic acid

**Figure 2:**
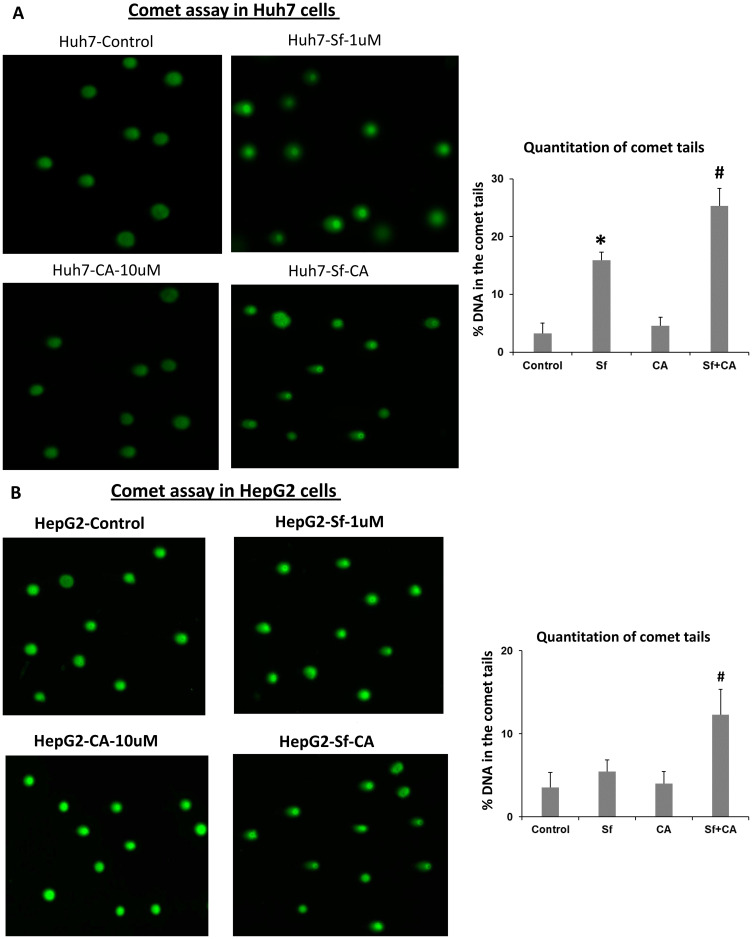
Comet assays of DNA damage in sorafenib and/or carnosic acid-treated HCC cells. Representative images of tailing by damaged DNA in Huh7 (**A**) and HepG2 (**B**) cells show that treatment with carnosic acid (CA) alone for 24 hours has no apparent effect on both cell lines (left-side panels). As expected, there is DNA damage when the cells are exposed to 1 μM sorafenib (Sf) alone, for 24 hours. DNA damage is increased when Sf is combined with CA (right-side panels). Quantitation of comet tails were shown in the bar charts, as described in Materials and Methods. ^*^ *p* < 0.05 *vs.* control; ^#^ *p* < 0.05 *vs.* Sf alone; *n* = 3.

The evidence of increased DNA damage in both HCC lines was obtained in several ways, including the Comet assay (Figure 2A and 2B). In the illustrative microscopic fields shown on the left-side panels of Figure 2 the “Comet tails”, i.e., the degraded DNA trailing behind the nuclei seen following electrophoresis, are enumerated. The percentages of the nuclei with tails are shown on the right-side panels of Figure 2.

Note that the evidence of DNA damage was observed in parallel with the increased ROS production (compare Figures 1 and 2) and that HepG2 cells demonstrate somewhat greater enhancement by CA of ROS generation induced by Sf alone (Figure 1) with a similar pattern demonstrated in the DNA damage assay (Figure 2).

**Figure 3:**
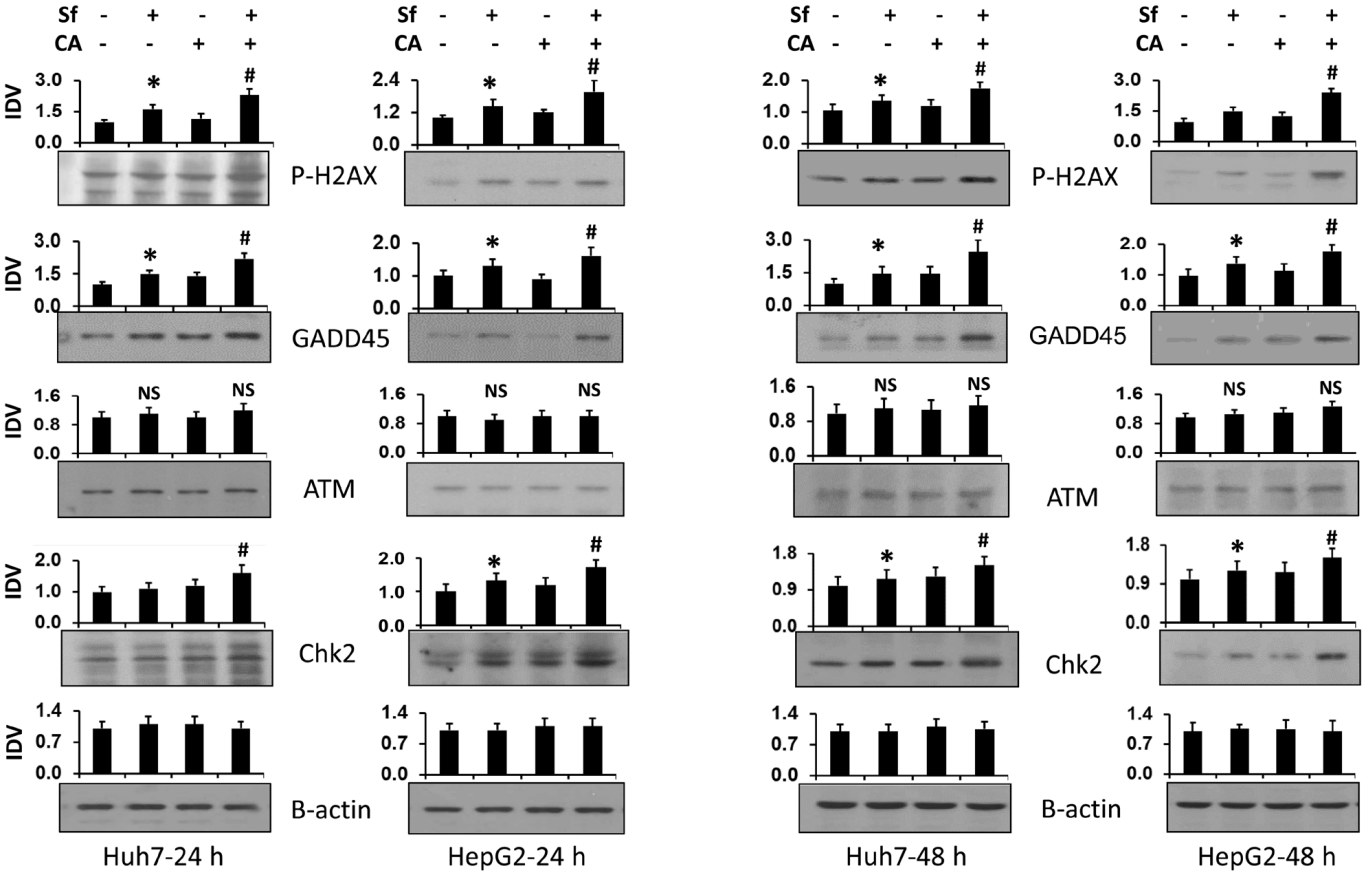
Combination of carnosic acid with sorafenib increases the expression of proteins related to DNA damage. Huh7 and HepG2 cells were treated with the indicated agents for 24 or 48 hours. The levels of proteins related to DNA damage, P-H2AX, GADD45, ATM, and Chk2, were determined by western blots. β-actin was used as the loading control. Average Integrated Density Values (IDV) from three separate experiments are shown in bar charts above each blot. ^*^ *p* < 0.05 *vs.* control; ^#^ *p* < 0.05 *vs.* Sf alone.

**Figure 4:**
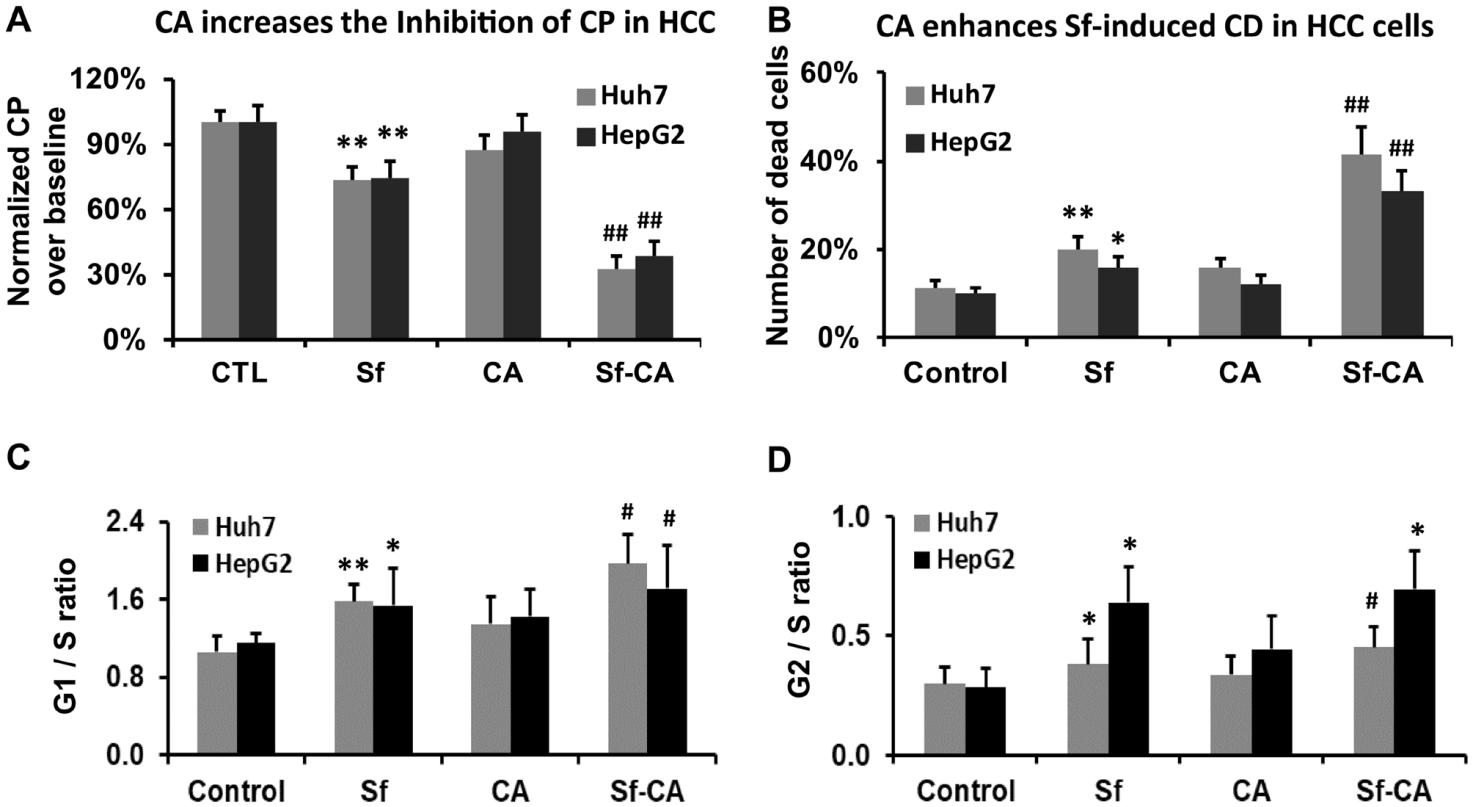
Carnosic acid enhances the sorafenib-induced retardation of cell proliferation and cell death. The total cell number of Huh7 and HepG2 cells and the number of dead cells were enumerated by the hemocytometer following treatment with the indicated agents for 24 hours, as detailed in Materials and Methods. (**A**) Cell proliferation (CP) was calculated as the percent of an increase in the total cell number over the baseline cell number relative to CP of the control sample. (**B**) The percent of dead cells was calculated as the number of trypan blue-positive cells relative to the total number of cells. (**C**, and **D**) Combination of CA (10 μM) and Sf (1 μM) significantly decreases cell proliferation and viability in both HCC cell lines. Cell cycle distribution was determined by Propidium Iodide staining, and the ratios of G1/S and G2/S are demonstrated in the bar charts. ^*^ *p* < 0.05; ^**^ *p* < 0.01 *vs.* control; ^#^ *p* < 0.05 *vs.* Sf alone; *n* = 3.

Additional evidence of DNA damage was demonstrated by the increased protein expression of the well-recognized markers of this damage, gamma-H2AX (P-H2AX) and Gadd45A (Figure 3). Although ATM showed no upregulation, ATR did (Figure 4). Since the evidence of DNA damage was already clear at 24 h, we limited the consequent studies to this early time period.

### Carnosic acid enhances the Sf-induced retardation of cell proliferation and cell death

Liver tumor growth, which this study was designed to model, depends on the rate of cell division as well as the rate of cell death. Here we explored if cell death and cell culture proliferation are regulated in tandem or independently, under the experimental conditions examined in this study. Therefore, we enumerated the total cell numbers, both viable and the non-viable, as a measure of cell proliferation, and we assessed cell death following single and combined treatments with Sf and CA. Figure 4 shows that in both HCC cell lines, cell proliferation (the total cell number visible in the hemocytometer) was significantly reduced by Sf alone, and markedly further reduced by the addition of CA, even though CA alone had a minimal, non-significant effect (Figure 4A). Also, cell death was increased with quite similar characteristics. Interestingly, the percentage loss of viability (i.e., cell death) in the group treated with Sf/CA and the percentage reduction of cell proliferation in this group were comparable, though reciprocal, in both cell lines.

### Cell cycle changes underlying the reduced proliferation rates

The basis for the CA-increased cell death in this experimental system have been already reported, as being due to both apoptosis and cytotoxic autophagy, with increased levels of the pro-apoptotic factor-BIM and cleaved Caspase 3, as well as Beclin1 and LC3-II, along with other molecular markers of cell death [13]. However, while it is known that Sf alone causes a retardation of cell cycle (CC) progression, the CA-induced enhancement of this Sf action on HCC cells has not been previously reported. Figure 4C and 4D shows that the addition of CA further increases the Sf-induced G1 prolongation, but the prolongation of G2/M by CA is not apparent.

**Figure 5:**
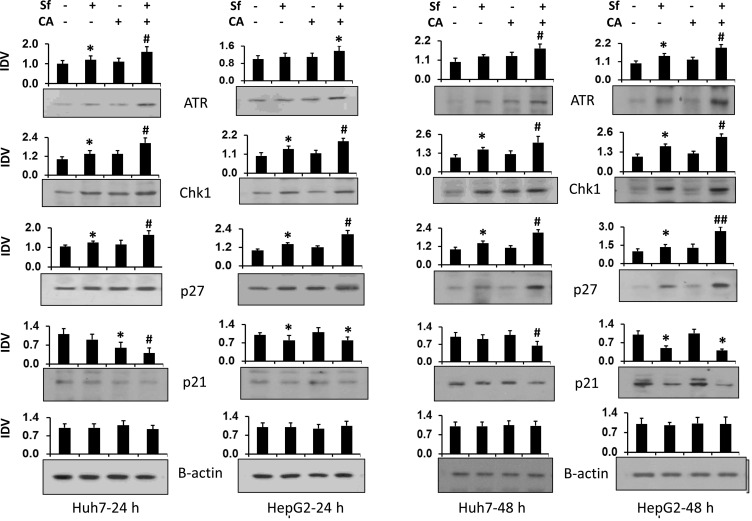
Carnosic acid enhances the sorafenib-induced increase in the expression of proteins related to DNA damage and cell cycle arrest. Following treatment of Huh7 and HepG2 cells with the indicated agents for 24 or 48 hours. The levels of proteins related to DNA damage (ATR and Chk1) and cell cycle progression inhibitors (p21 and p27) were determined by western blots. β-actin was used as the loading control. Average Integrated Density Values from three separate experiments are shown in the bar charts above each blot. ^*^ *p* < 0.05 *vs.* control; ^#^ *p* < 0.05 *vs.* Sf alone.

The determination of the levels of proteins which can transmit DNA damage to cell CC arrest, ATR and its downstream target Chk1 [21–23], as well as the inhibitor of CC progression p27/Kip1 (p27) [24–26], shows their elevation by Sf as expected (Figure 5). In addition, the CA enhancement was also seen, though the increases were close to the limits of the accuracy of the measurements of band density. Two time points were studied to confirm that these proteins participated in the CC regulation when CA was added to Sf. Interestingly, the levels of another inhibitor of the CC progression p21/Cip1 (p21) were decreased, providing a negative control for the upregulation of ATR, Chk1, and p27 (Figure 5).

### Upstream regulators participating in the enhancement of cell growth inhibiting effects of the Sf/CA combination

**Figure 6:**
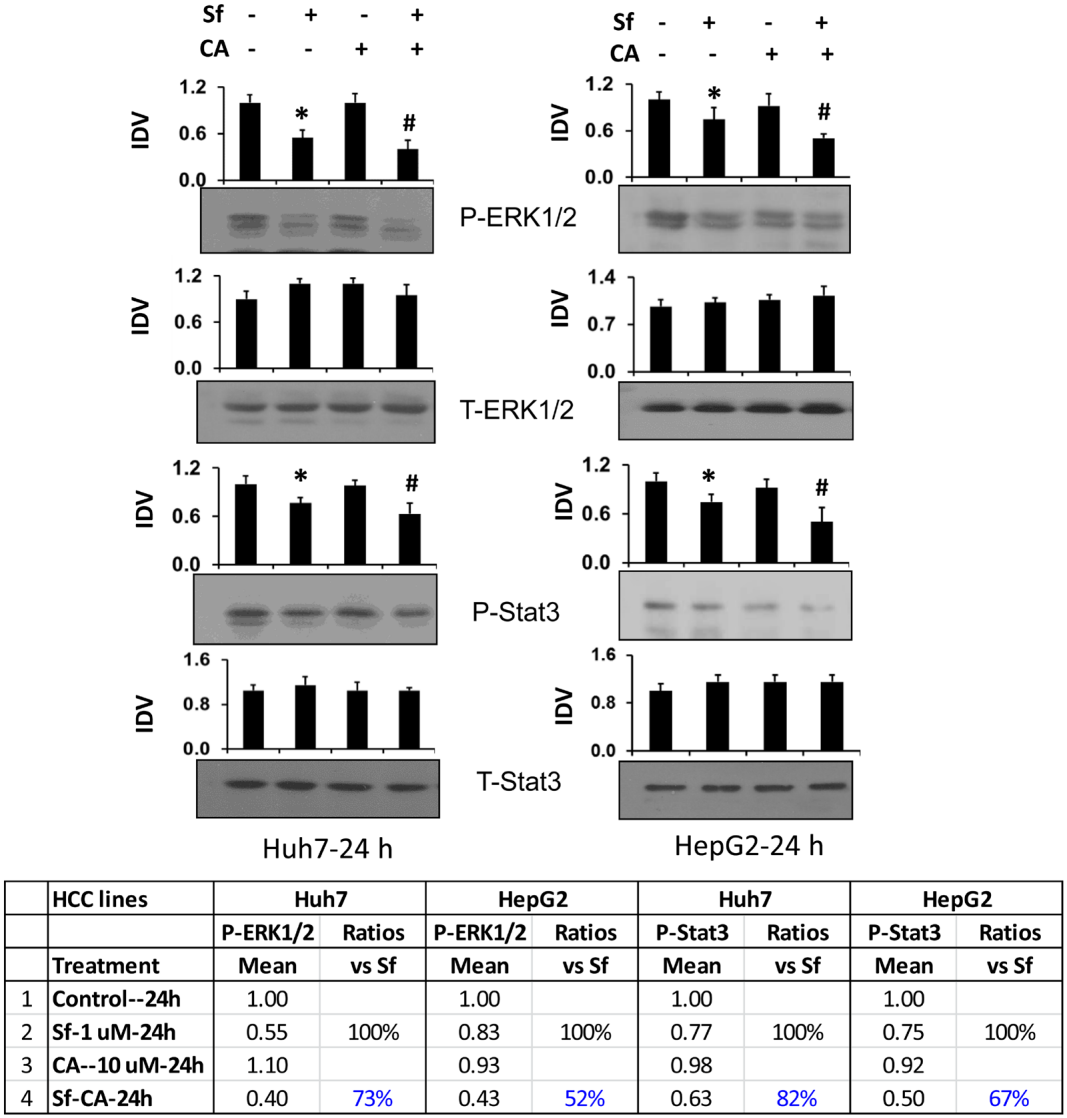
Carnosic acid enhances the sorafenib-induced reduction of P-ERK1/2 and P-STAT3 levels in HCC cells. Huh7 and HepG2 cells were treated with the indicated agents for 24 hours. The protein levels of P-ERK1/2 (Thr202/Tyr204), P-STAT3 (Tyr605), and total (T) ERK1/2 and STAT3 were determined by western blots. The ratios of P-ERK1/2 *vs.* T-ERK1/2 and P-STAT3 *vs.* T-STAT3 are shown in the table below. CA further enhances Sf-induced reduction of activation of both ERK1/2 and STAT3.

It appears likely that extracellular signals such as cytokines (e.g., [27]) and the actions of Sf other than DNA damage can also activate the executioner machineries for the cell death and cell proliferation regulation observed here. Some such upstream regulators, including ERK1/2 and STAT3, have been reported to be a part of Sf action in various neoplastic cells [28–31]. In view of the widely known functions of these regulators in cell proliferation and cell survival, we performed western blot analyses of the levels of these proteins in HCC cells treated with Sf, CA, and their combination. Figure 6 shows that activating phosphorylations of both ERK1/2 and STAT3 are reduced by Sf and become significantly lower when CA is added to Sf, although the total levels of these proteins are unchanged. This results in the ERK1/2 and STAT3 activation ratio in Sf/CA-treated cells becoming lower than that in cells treated with Sf alone, consistent with a lower activity of ERK1/2 and STAT3 in the combination condition versus Sf alone.

In order to determine if the observed changes in the phosphorylation levels of ERK1/2 and STAT3 have a functional significance, we used pharmacological inhibitors of these proteins. We then determined the effects of the inhibitors on cell death, cell proliferation, and phosphorylation of these proteins and on the levels of proteins that mark DNA damage, CC progression as well as some selected contributors to the execution of apoptosis and autophagy. As shown in Tables 1A and 2A, cell death was not significantly inhibited in the otherwise untreated HCC cells of both cell lines by the reduction of P-ERK1/2 activity by U0126, an inhibitor of the kinase activity of MEK1 (Tables 1A and 2A, column 6, group 5 [G5] *vs.* group 1 [G1]). However, U0126 did significantly diminish the reduction in the cell number increase (i.e., cell proliferation) (Tables 1B and 2B, column 2, G5 *vs.* G1). Conversely, when the untreated cells were exposed to the specific STAT3 inhibitor Stattic (Tables 1 and 2, column 1, G9-G1), cell death was moderately but significantly inhibited in HepG2 cells, and a similar percentage of cell proliferation reduction was observed in Huh7 cells, though the decrease did not reach significance in Huh7 cells. These data suggest that the cell proliferation of HCC cells in culture primarily requires an optimal activity of ERK1/2, and cell death requires the activity of STAT3.

Accordingly, we examined the dependence of the effects of Sf, alone or in combination with CA, on the phosphorylation of ERK1/2 and STAT3. Group comparisons in Sf alone-treated Huh7 cells showed that the functionality of the above regulators was very similar to those described above in untreated Huh7 cells, with significant changes in cell death only in the STAT3-inhibited (Table 1A, column 7, G6 *vs.* G2) and in cell proliferation in the ERK1/2-inhibited cells (Table 1B, G6 *vs.* G2). However, in HepG2 cells, both Stattic and U0126 had significant effects, though the effect of STAT3 inhibition (Table 2A, G10 *vs.* G2) on cell death was greater than of ERK1/2 inhibition (Table 2A, G6 *vs.* G2), while ERK1/2 inhibition had a greater effect on the inhibition of cell proliferation (Table 2B, G6 *vs.* G2).

**Table 1:** Effects of ERK1/2 and STAT3 inhibitors on cell death (A) and cell proliferation (B) in Huh7 cells

A.		Cell death (CD) and) and supra-additivity of Sf and CA effects					Comparisons between treatment groups			
	Huh7	1. Mean	2. SD	3. Net	4. *P* values	5.	6. Comparison	7. Comparison	8. Comparison	9. Comparison
	*n* = 6	CD	CD	CD	vs Control	Supra-additivity	vs Control	vs Sf only	vs CA only	vs Sf+CA
**1**	**Control-24 hr**	**11.3%**	1.5%	**vs G1**	**vs G1**	**Based on net CD**		**G1–G2 = –8.7%**		
**2**	**Sf-1 uM**	**20.0%**	2.6%	8.7%	0.002	**G4–(G2+G3)**		*p* = 0.002		
**3**	**CA-10 uM**	**15.7%**	2.1%	4.4%	0.001	30.0%–(4.4%+8.7%)				
**4**	**Sf-CA**	**41.3%**	3.5%	30.0%	0.001	**17.0%**				
**5**	**U0126-1 uM**	**11.7%**	2.3%	**vs G5**	**vs G5**		**G5–G1 = 0.4%**	**G6–G2 = –1.3%**		
**6**	**U0126-Sf**	**18.7%**	2.5%	7.0%	0.007	**G8–(G6+G7)**	*p* = 0.477	*p* = 0.382	**G7–G3 = 2.0%**	
**7**	**U0126-CA**	**16.7%**	2.1%	5.0%	0.025	23.6%–(7.0%+5.0%)			*p* = 0.111	**G8–G4 = –6.0%**
**8**	**U0126-Sf-CA**	**35.3%**	4.5%	23.6%	0.004	**11.7%**				*p* = 0.064
**9**	**Stattic-20 uM**	**15.7%**	3.1%	**vs G9**	**vs G9**		**G9–G1 = 4.4%**	**G10–G2 = 14.3%**		
**10**	**Stattic-Sf**	**34.3%**	3.1%	18.6%	0.004	**G12–(G10+G11)**	*p* = 0.058	*p* = 0.016	**G11–G3 = 11.2%**	
**11**	**Stattic-CA**	**29.7%**	1.5%	14.0%	0.002	35.3%–(18.6%+14.0%)			*p* = 0.001	**G12–G4 = 9.7%**
**12**	**Stattic-Sf-CA**	**51.0%**	4.4%	35.3%	0.001	**2.7%**				*p* = 0.020
**B.**		**Cell proliferation (CP) and ) and supra-additivity of Sf and CA effects**					**Comparisons between treatment groups**			
	**Huh7**	**1. Cell number**	**2. Increase**	**3. Increase**	**4. *P* values**	**5.**	**6. Comparison**	**7. Comparison**	**8. Comparison**	**9. Comparison**
	**Base = 136k/mL, *n* = 6**	**Mean (k/mL)**	**over Base**	**SD**	**vs CTL**	**Supra-additivity**	**vs Control**	**vs Sf only**	**vs CA only**	**vs Sf+CA**
**1**	**Control-24 hr**	**207.1**	**52.3%**	5.5%	**vs G1**	**Based on % increase**		**G1–G2 = –13.5%**		
**2**	**Sf-1 uM**	**188.8**	**38.8%**	6.5%	0.003	**G4–(G2+G3)**		*p* = 0.003		
**3**	**CA-10 uM**	**198.8**	**46.1%**	6.5%	0.129	18.6%–(38.8%+46.1%)				
**4**	**Sf-CA**	**161.3**	**18.6%**	6.9%	0.002	**–66.4%**				
**5**	**U0126-1 uM**	**180.4**	**32.7%**	5.7%	**vs G5**		**G5–G1 = –19.6%**	**G6–G2 = –27.0%**		
**6**	**U0126-Sf**	**152.1**	**11.8%**	10.1%	0.003	**G8-(G6+G7)**	*p* = 0.001	*p* = 0.0002	**G7–G3 = –21.4%**	
**7**	**U0126-CA**	**169.6**	**24.7%**	7.1%	0.003	3.9%–(11.8%+24.7%)			*p* = 0.001	**G8–G4 = –14.7%**
**8**	**U0126-Sf-CA**	**141.3**	**3.9%**	5.5%	0.002	**–32.7%**				*p* = 0.002
**9**	**Stattic-20 uM**	**213.8**	**57.2%**	8.4%	**vs G9**		**G9-G1 = 4.9%**	**G10–G2 = 1.8%**		
**10**	**Stattic-Sf**	**191.3**	**40.6%**	6.5%	0.008	**G12–(G10+G11)**	*p* = 0.287	*p* = 0.2939	**G11–G3 = –14.7%**	
**11**	**Stattic-CA**	**178.8**	**31.4%**	6.9%	0.001	11.8%–(40.6%+31.4%)			*p* = 0.002	**G12–G4 = –6.7%**
**12**	**Stattic-Sf-CA**	**152.1**	**11.8%**	4.8%	0.004	**–60.2%**				*p* = 0.012

Huh7 cells. (**A**) The percent of dead cells (CD) was determined by the trypan blue exclusion assay (column 1). The net CD was calculated as the difference between each treatment group and the corresponding control group (column 3). (**B**) Cell proliferation (CP) was determined as the percent of the total cell number increase over the baseline cell number (column 2). Supra-additivity was calculated in both A and B as the difference between the effect of the Sf/CA combination and the sum of the effects of Sf and CA alone (column 5). The results of the comparison between different control and treatment groups are presented in columns 6–9. The data are means ± SD of three independent experiments performed in triplicate, *n **=*** 9.

**Table 2:** Effects of ERK1/2 and STAT3 inhibitors on cell death (A) and cell proliferation (B) in HepG2 cells

A.		Cell death (CD) and supra-additivity of Sf and CA effects					Comparisons between treatment groups			
	HepG2	1. Mean	2. SD	3. Net	4. *P* values	5.	6. Comparison	7. Comparison	8. Comparison	9. Comparison
	*n* = 9	CD	CD	CD	vs Control	Supra-additivity	vs Control	vs Sf only	vs CA only	vs Sf+CA
1	**Control--24 hr**	**9.9%**	3.0%	**vs G1**	**vs G1**	**Based on net CD**		**G1–G2 = –6.0%**		
2	**Sf-1 uM**	**15.9%**	3.1%	6.0%	0.013	**G4–(G2+G3)**		*p* = 0.013		
3	**CA-10 uM**	**12.2%**	2.6%	2.3%	0.059	23.3%–(6.0%+2.3%)				
4	**Sf-CA**	**33.2%**	4.6%	23.3%	0.001	**15.0%**				
5	**U0126-1 uM**	**11.8%**	3.7%	**vs G5**	**vs G5**		**G5–G1 = 1.9%**	**G6–G2 = 3.8%**		
6	**U0126-Sf**	**19.7%**	3.6%	7.9%	0.012	**G8–(G6+G7)**	*p* = 0.265	*p* = 0.018	**G7–G3 = 6.2%**	
7	**U0126-CA**	**18.4%**	3.9%	6.6%	0.001	21.9%–(7.9%+6.6%)			*p* = 0.002	**G8–G4 = 0.4%**
8	**U0126-Sf-CA**	**33.7%**	3.5%	21.9%	0.001	**7.4%**				*p* = 0.836
9	**Stattic-20 uM**	**14.2%**	3.2%	**vs G9**	**vs G9**		**G9–G1 = 4.3%**	**G10–G2 = 11.9%**		
10	**Stattic-Sf**	**27.8%**	3.2%	13.6%	0.001	**G12–(G10+G11)**	*p* = 0.015	*p* = 0.001	**G11–G3 = 11.2%**	
11	**Stattic-CA**	**23.4%**	4.0%	9.2%	0.002	34.4%–(13.6%+9.2%)			*p* = 0.001	**G12–G4 = 14.9%**
12	**Stattic--Sf-CA**	**48.6%**	4.1%	34.4%	0.003	**11.5%**				*p* = 0.004
**B.**		**Cell proliferation (CP) and supra-additivity of Sf and CA effects**					**Comparisons between treatment groups**			
	**HepG2**	**1. Cell number**	**2. Increase**	**3. Increase**	**4. *P* values**	**5.**	**6. Comparison**	**7. Comparison**	**8. Comparison**	**9. Comparison**
	**Base = 148k/mL, *n* = 7**	**Mean (k/mL)**	**over Base**	**SD**	**vs CTL**	**Supra-additivity**	**vs Control**	**vs Sf only**	**vs CA only**	**vs Sf+CA**
1	**Control-24 hr**	**240.7**	**62.6%**	9.2%	**vs G1**	**Based on % increase**		**G1–G2 = –14.5%**		
2	**Sf-1 uM**	**219.3**	**48.2%**	10.8%	0.004	**G4–(G2+G3)**		*p* = 0.004		
3	**CA-10 uM**	**237.1**	**60.2%**	7.8%	0.937	23.3%–(48.2%+62.6%)				
4	**Sf-C**	**182.5**	**23.3%**	7.5%	0.006	**–85.1%**				
5	**U0126-1 uM**	**217.1**	**46.7%**	6.3%	**vs G5**		**G5–G1 = –15.9%**	**G6–G2 = –25.6%**		
6	**U0126-Sf**	**181.4**	**22.6%**	8.0%	0.002	**G8–(G6+G7)**	*p* = 0.005	*p* = 0.002	**G7 -G3 = -36.3%**	
7	**U0126-CA**	**184.6**	**24.8%**	8.0%	0.002	3.0%–(22.6%+24.8%)			*p* = 0.001	**G8–G4 = –20.9%**
8	**U0126-Sf-CA**	**152.5**	**3.0%**	7.7%	0.001	**–44.3%**				*p* = 0.001
9	**Stattic-20 uM**	**241.8**	**63.4%**	10.5%	**vs G9**		**G9–G1 = 0.7%**	**G10–G2 = –14.0%**		
10	**Stattic-Sf**	**198.6**	**34.2%**	6.6%	0.001	**G12–(G10+G11)**	*p* = 0.927	*p* = 0.009	**G11–G3 = –32.7%**	
11	**Stattic-CA**	**190.0**	**28.4%**	5.3%	0.001	1.1%–(34.2%+28.4%)			*p* = 0.001	**G12–G4 = –23.1%**
12	**Stattic-Sf-CA**	**149.6**	**1.1%**	5.4%	0.001	**–61.5%**				*p* = 0.001

HepG2 cells. (**A**) The percent of dead cells (CD) was determined and calculated as detailed in the legend to Table 1. (**B**) Cell proliferation (CP) was determined and calculated as detailed in the legend to Table 1. The data are means ± SD of three independent experiments performed in triplicate, *n **=*** 9.

When CA was added to Sf, ERK1/2 or STAT3 inhibition in both cell lines resulted in a marked potentiation of the cell proliferation-inhibitory effect of the combined treatment, indicating that both ERK1/2 and STAT3 protect HCC cells from the antiproliferative effect of the Sf/CA combination (Tables 1B and 2B, columns 9). However, the ERK1/2 inhibitor did not affect Sf/CA-induced cell death in either cell line (Tables 1A and 2A, columns 9), but STAT3 inhibition significantly increased cell death in both cell lines (Tables 1B and 2B, columns 9), suggesting that STAT3 can also protect HCC cells from Sf/CA-induced cytotoxicity.

Regarding the supra-additive effects of adding CA to Sf, the data show that both ERK1/2 and STAT3 are needed for the maximal interactive synergy between the two agents in inducing cell death (Tables 1A and 2A, columns 5). ERK1/2 appears to be required for the supra-additive cell proliferation inhibition in both cell lines, while STAT3 contributes to the supra-additivity in HepG2 cells but has a minimal, if any, effect in Huh7 cells on cell proliferation (Tables 1B and 2B, columns 5).

**Figure 7:**
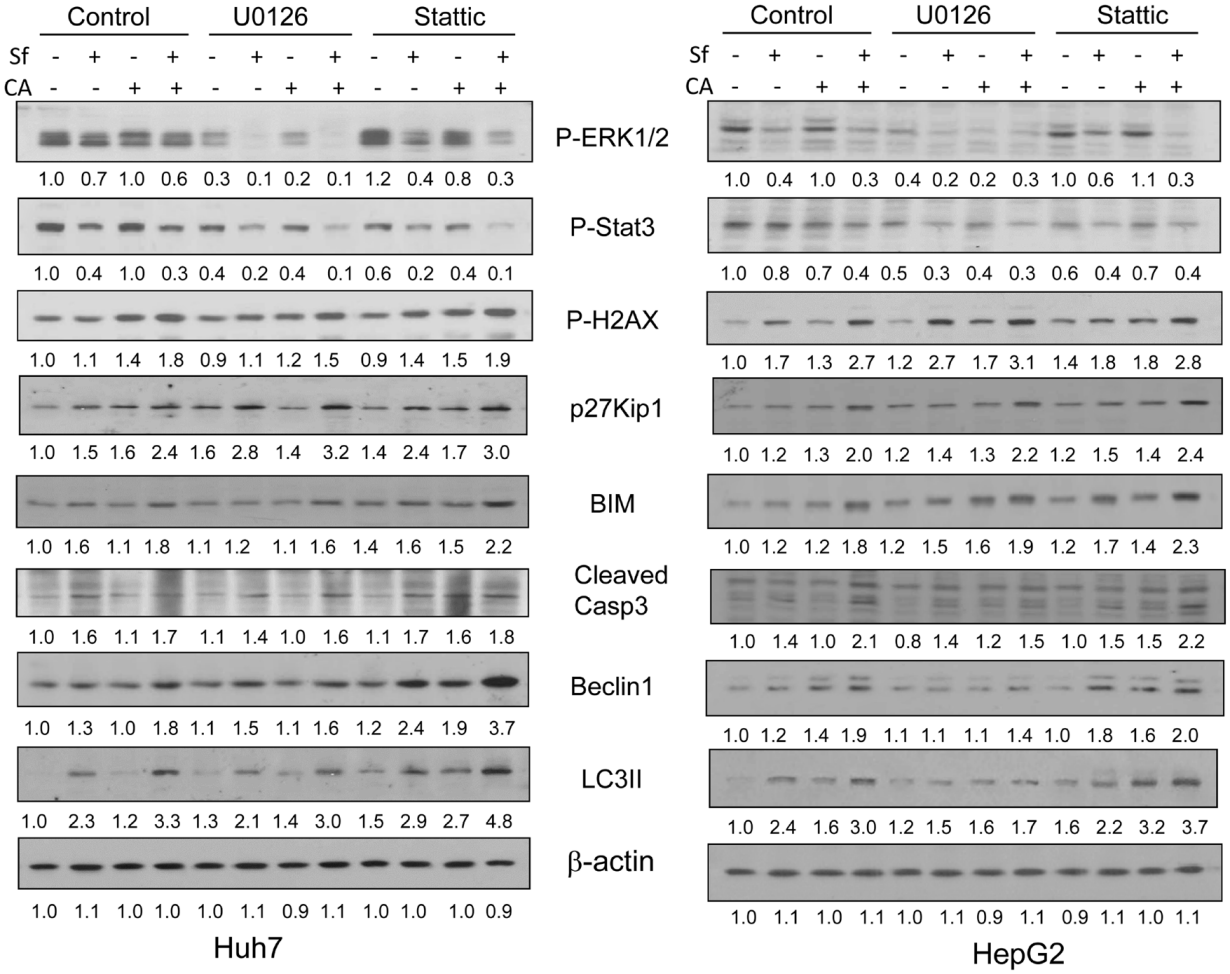
The effects of inhibition of ERK1/2 or STAT3 by pharmacological inhibitors on cell death and cell cycle related proteins. HCC cells were pretreated with either MEK1 inhibitor U0126 (1 μM) or the STAT3 inhibitor Stattic (20 μM) for 1 hour, and then treated with indicated agents for another 24 hours. The protein levels of P-ERK1/2, P-STAT3, and cell death- and cell cycle-related targets were determined by western blotting. β-actin was used as the loading control. The blots shown illustrate one of three individual experiments, and Integrated Density Values are shown under each blot.

The overall conclusion from these experiments is that while there is a clear overlap in the functionality of ERK1/2 and STAT3 in HCC cells, the former is essentially a regulator of cell proliferation [32] and the latter of cell death [33]. To support this conclusion, we performed western blot studies of several relevant regulatory proteins. Initially, we tested the effectiveness of ERK1/2 inhibition, and found that in otherwise untreated cells of both cell lines ERK1/2 activation was reduced by 50–70%, while STAT3 inhibitor depleted P-STAT3 levels by about 40%. Surprisingly, ERK1/2 inhibition also resulted in reduced P-STAT3 levels in both cell lines, but was more marked and statistically significant, in Huh7 cells (Figure 7 and Supplementary Tables 1 and 2). The latter finding suggests that ERK1/2 is an upstream regulator of STAT3 and thus explains the overlap in their actions on cell proliferation, even though this overlap is incomplete. Sf, with and without CA, further reduced P-ERK1/2 and P-Stat levels, but the signals in the immunoblots became too low for an accurate analysis.

The levels of gamma-H2AX in both cell lines were increased by Sf, CA, and Sf/CA, and these increases were amplified by both ERK1/2 and STAT3 inhibitors, primarily in HepG2 cells, though the significance of this difference is uncertain. Amplification of p27 expression by ERK1/2 and STAT3 inhibitors was seen in both cell lines, suggesting that both ERK1/2 and STAT3 protect HCC cells from Sf/CA-induced inhibition of cell cycle.

Both Sf alone and Sf/CA induced increases in the apoptosis molecular markers Bim, cleaved Caspase 3 and in the autophagy markers Beclin1 and LC3. These increases in markers were moderately but quite consistently amplified by STAT3 inhibition but not by ERK1/2 inhibition (Figure 7 and Supplementary Tables 1 and 2). Again, these data support the conclusion that ERK1/2 activity is primarily a regulator of cell proliferation, and STAT3 of cell death, in HCC cells treated with the Sf/CA combination.

## DISCUSSION

Most of the chemo-therapeutic agents currently in clinical use which have shown some effects on the progression of high mortality malignancies, as for example cytosine arabinoside (AraC) for acute myeloid leukemia (AML), or Sorafenib for HCC, are regarded as primarily cytotoxic drugs [1, 34]. This implies that cell death, usually attributed to apoptosis, is the main mechanism responsible for the therapeutic effect. However, it seems clear that cell cycle (CC) arrest or the retardation of its progression are also factors [35–37]. In laboratory studies, predominantly in tissue or cell culture, the availability of dyes, such as MTT or WST-8, which easily provide machine readings of the relative changes in the quantity of the viable cells have made a visual enumeration of the cells (whether viable, persisting apoptotic, or necrotic cells) or “nearly obsolete”. Yet to understand the mechanisms which underlie tumor growth, a separation of cell proliferation and cell death is essential, since cell death executioner machinery and CC control effectors are well known to be unrelated. Thus, the main novelty of this study is the demonstration that the upstream controls of malignant cell growth can have varying, separable effects on both CC and cell death. We show this by selecting the transcription factor activating kinase ERK1/2 and the transcription factor STAT3 as models for upstream regulation of HCC tumor cell growth.

The secondary objective of this study was to determine the sequence of molecular events that result in the enhanced cell death and the retardation of CC progression when carnosic acid (CA), a plant-derived mild anti-oxidant [12], is added together with Sorafenib (Sf) to human HCC cells, an observation we have recently reported [13]. Since, when applied alone, Sf is known to increase the generation of intracellular ROS, and this seems to be an early event in the cytotoxic action of Sf [14, 15], we determined cytosolic ROS levels and confirmed that these increase following the addition of a low concentration of Sf, and are then further elevated when CA is added to Sf (Figure 1). It has been reported that at high concentrations (30–60 μM) CA alone induces ROS generation in HCC cells [38], suggesting that CA may synergistically interact with Sf and thus becomes capable of potentiating Sf-induced ROS production, even at its lower concentration of 10 μM.

Other polyphenols have also been shown to potentiate the *in vitro* anticancer effects of Sf on various tumor cell types (e.g., [10, 11, 39, 40]). The effects of such combinations on the intracellular ROS levels were scarcely studied. However, the enhanced apoptosis induction by the Sf/resveratrol combination in MCF-7 breast cancer cells [39] or by the Sf/oleanolic acid combination in Huh7 and HepG2 cells [41] was found to be associated with augmented ROS generation. While there is a correlation between the cooperative elevation of ROS levels by Sf/CA and the subsequent events studied here that lead to enhanced cell death and retardation of CC progression, the mechanistic elucidation of this correlation will require additional extensive studies of the roles of ROS generation, redox-related signaling pathways, and transcription factors.

Sf is known to generate DNA damage among its other pleiotropic actions [42] perhaps by generating ROS [14, 43]. We demonstrate here by the comet assay (Figure 2) and by western blotting with molecular markers (Figure 3), that CA markedly enhances DNA damage. This becomes apparent at 24 h, suggesting that this is an early event in the CA enhancement of Sf action. The potential consequences of DNA damage include a CC arrest, and as expected, the CA-increased damage was also reflected by an increase in Sf- induced retardation of the G1 to S transition (Figure 4C). Although Sf also causes a G2/M arrest, this was not apparent as a part of the CA addition to Sf (Figure 4D). Similarly, in Sf-treated cells we consistently observed an increased expression of Chk1 and Chk2, proteins which control the CC check points and are regulated by the DNA damage sensor and transducer ATR, and the expression of both was increased when CA was added (Figure 5). However, we did not see any effect of Sf or CA on ATM, another DNA damage and CC-related protein, showing the selectivity of CC regulation, also exemplified by the downregulation of p21 (Figures 3 and 5).

Other studies have demonstrated that the cooperative effects of Sf/polyphenol combinations on ERK1/2 and STAT3 in HCC cells; differ depending on the type of phenolic compound. Thus, combined treatments with Sf and silibinin or the curcumin analog ASC-J9r were shown to cooperatively reduce phosphorylation of ERK1/2 and/or STAT3 [11, 44] while the Sf/chrysin combination induced sustained ERK1/2 phosphorylation [45]. Moreover, overexpression of Mek1 enhanced, whereas inhibition of Mek1 by U0126 reduced the synergistic reduction of cell viability by Sf/chrysin [45]. It is known that ERK1/2 phosphorylates STAT3 at Ser727 in human bladder cells and that STAT3 plays the key role directly downstream from ERK1/2 as an alternative survival pathway in neurons [46, 47]. However, while ERK1/2 and STAT3 have been studied in relation to the enhancement of Sf action in many cell types, their relative roles in the control of cell proliferation and cell death in any cell type have previously been unclear.

Two HCC cell lines were studied here to identify features likely to have some general significance, and most of our results re-enforced one another. However, HepG2 cells exhibit greater association between the CA enhancement of Sf-induced ROS production and DNA damage, as detected by the Comet assay. Further, in HepG2 cells the levels of P-H2AX were amplified by ERK1/2 and STAT3 inhibitors to a greater extent than in Huh7 cells. These differences may be related to the origin of HepG2 cells derived from a young patient with hepatoblastoma [48], while Huh7 cells were cultured from a hepatocellular carcinoma [49], and thus the former are arrested at a more advanced state of cell differentiation. However, a larger range of HCC lines needs to be examined in order to assign these differences to any particular cause.

We have previously reported that CA enhanced Sf-induced cell death by both apoptosis and cytotoxic autophagy [13], but it is possible that other forms of cell death such as pyroptosis, which can further activate the innate immune system [50]. This may contribute to the Sf/CA-induced cell death of HCC *in vivo* and will be an area for interesting future studies. Also, a caveat that cell death contributes to the loss of cell numbers apparent in this study is possible, though we reduced the extent that this may be happening by focusing on the relatively short treatment period, the first 24 hours. This may also relate to the apparent expansion of the G1 compartment in our cell cycle studies but can also reflect differential loss of cells in different cell cycle phases, a possible avenue for future studies.

In order to increase any potential clinical significance of these studies, perhaps even before the replication in animal models, additional *in vitro* studies, both mechanistic and further preclinical, will be needed. The evidence we provide here suggests that a combination of three agents, Sf, CA, and Stattic may be more clinically effective, but these may be further enhanced by adding an inhibitor of other survival-promoting factors, such as NF- kB or PI3K/Akt, as some malignancies such as Hodgkin’s require quintuple poly-chemotherapy [51]. These topics are projected as extensive future endeavors.

## MATERIALS AND METHODS

### Chemicals and antibodies

Sorafenib (Sf) was purchased from Millipore-Sigma (St. Louis, MO, USA), and it was used at the final concentration of 1 μM. Carnosic acid (CA) was purchased from Enzo Life Sciences, Inc., (Farmingdale, NY, USA) and used at the final concentration of 10 μM [13, 20]. Probenecid and 2′,7′-dichlorodihydrofluorescein diacetate (DCFH-DA) were obtained from Merck-Sigma-Aldrich. The MEK1 inhibitor U0126 was purchased from Cell Signaling Inc., (Danvers, MA, USA) and the STAT3 inhibitor Stattic from Selleck Chemicals (Houston, TX, USA). The antibodies purchased from Cell Signaling Technologies (Danvers, MA, USA) include the following: Phospho-specific antibodies against ERK1/2 (Thr202/Tyr204), STAT3 (Tyr605, #52075), H2AX (Ser139, #9718); corresponding specific antibodies against ERK1/2 (#9102), STAT3 (#4904), H2AX (#2595), Bim (#2819), Cleaved Caspase-3 (#9661), Chk1 (#2360), Chk2 (#2662), ATM (#2873), ATR (#2790), p21Cip1 (#2947), p27Kip1 (#3688), and HRP-linked anti-rabbit (#7074). The β-actin antibody was obtained from Merck-Sigma-Aldrich. Propidium Iodide Nucleic Acid Stain kit was from Invitrogen (Carlsbad, CA, USA).

### Cells and culture

Two human HCC cell lines were used in this study: Huh7 and HepG2 cells. These cells were cultured in Dulbecco’s Modified Eagle medium (Gibco, Gaithersburg, MD, USA) supplemented with 10% heat-inactivated fetal calf serum (Thermo Fisher Scientific, Waltham, MA, USA), 2 mM glutamine, 1% penicillin/streptomycin at 37°C in a 5% CO_2_ environment. The cells were passaged twice a week to maintain log phase growth. In preliminary experiments the dosage of the compounds used was determined by *in vitro* cytotoxicity evaluation. The cells were exposed to a range of concentrations of a compound for 24–48 hours to determine the IC50 which was then used in the experiments. For most experiments, the cells were seeded in 6-well plates at a density of 1.0 × 10^5^/mL and grew to about 50% confluency before incubation with experimental agents. The baseline cell number was determined at this point for the calculation of cell proliferation rates. The results are presented in the following sequence: (i) Addition of vehicle (0.01% ethanol), the untreated control; (ii) 1 μM sorafenib alone; (iii) 10 μM CA; (iv) 1 μM Sorafenib in combination with 10 μM CA. For the inhibition of MEK signaling pathway and STAT3 transcription factor activity the additional groups were sequenced: (v) 1 μM U0126; (vi) 1 μM U0126 with 1 μM sorafenib; (vii) 1 μM U0126 with 10 μM CA; (viii) 1 μM U0126 with 1 μM Sorafenib and 10 μM CA; (ix) 20 μM Stattic; (x) 20 μM Stattic with 1 μM sorafenib; (vii) 20 μM Stattic with 10 μM CA; (iv) 20 μM Stattic with 1 μM Sorafenib and 10 μM CA.

### Determination of intracellular levels of reactive oxygen species

The intracellular ROS levels were determined using the oxidation-sensitive fluorescent indicator DCFH-DA, as described previously [16, 18], with modifications. Vehicle control and treated cells (5 × 10^5^) were harvested by trypsinization, washed with Hanks’ Balanced Salt solution (HBSS) containing 10 mM HEPES (pH = 7.4) and loaded with 5 μM DCFH-DA in HBSS/HEPES buffer containing 0.6 mM probenecid (Buffer A), for 15 min at 37°C in a shaking water bath. A parallel vehicle control sample was incubated under the same conditions in the absence of DCFH-DA and used for measuring background fluorescence. Cells were then washed with Buffer A and resuspended in the same buffer followed by flow cytometric analysis. For the positive control, DCFH-DA loaded vehicle-treated cells were washed and incubated with 500 μM H_2_O_2_ in Buffer A for an additional 30 min. The fluorescence intensity of the DCFH-DA oxidized product dichlorofluorescein (DCF) was measured in a Gallios flow cytometer (Beckman Coulter Inc.). For each analysis 10,000 events were recorded. The data were analyzed using Kaluza Analysis Software version 2.1.1 (Beckman Coulter).

### Quantitation of cell proliferation and death by trypan blue exclusion

To determine the total (live plus dead) cell number, trypsinized HCC cells were resuspended in 1 mL PBS and 20 μL of suspended cells were pipetted into counting chamber for enumeration without the addition of trypan blue. Cell proliferation was determined as the percent of the total cell number increase over the baseline cell number as follows: (Total cell number-Baseline cell number)/Baseline cell number. To determine the percentage of cell death, HCC cells were incubated with 0.4% trypan blue for 5 min and then 100 cells were counted in triplicate by hemocytometer for the number of dead cells.

### Comet assays of DNA damage

DNA damage in experimental HCC cells was measured by the comet assay kit from Cell Biolabs (San Diego, CA, USA), according to the manufacturer’s recommended protocol (Cell Biolabs). Briefly, Huh7 or HepG2 cells were trypsinized and washed twice with 1 × PBS, then 2,000 cells were mixed with low melting-point agarose gel and pipetted on the “Comet Slide”. The cells transferred to the slide were maintained for 15 min at 4°C in the dark, then immersed in the lysis buffer for 30 min at 4°C in the dark, to relax and denature the nuclear DNA. Next, the slides were transferred into a horizontal electrophoresis tank in TBE buffer, and ran for 15 min at 30 V. The slides were rinsed three times with distilled water and once with 70% ethanol. Finally, the dried slide was stained with Vista Green DNA Dye for 15 min at room temperature. The cells on the slide were visualized and photographed using a fluorescent microscope at the Rutgers Digital Imaging Core Facility. The images were analyzed with the semi-automated comet analysis software, Opencomet, which was used as a plugin for the image processing platform ImageJ [52]. All cells on the photographed images were analyzed by measuring the stained DNA tail intensity and total stained DNA intensity. Only the intensity profiles of the comets that were not in clumps or at the edges of the slide were scored. DNA damage was reported as the percentage of the DNA in comet tail.

### Cell cycle analysis

To evaluate DNA content in HCC cell cultures, 1 × 10^6^ cells were fixed in 75% ethanol at –20°C for at least 24 h. After washing twice with 1× PBS, the cells were incubated with 0.5 ml of PI-staining buffer (PBS with 100 μg/mL RNase A, 50 μg/mL Propidium Iodide) for 2 h at 4°C. The DNA content was determined using the EPICS XL Flow cytometer (Beckman Coulter Inc., Fullerton, CA, USA), and cell cycle distribution was analyzed by the Multicycle software package (Phoenix Flow Systems, San Diego, CA, USA).

### Western blotting

Whole cell lysates were prepared as follows: In each experiment the HCC cells were washed with ice-cold 1× PBS once and then lysed with RIPA buffer (Sigma, Cat#R0278) supplemented with Complete™ Protease Inhibitor Cocktail (Roche, Cat#11697498001), followed by centrifugation at 16,000 g for 30 min. The protein concentration of whole cell extracts was determined using Bio-Rad (Hercules, CA, USA) protein assay kit.

Western blotting was performed as previously described [53]. Briefly, whole cell lysates (15 μg protein) were separated on the SDS-PAGE gel and transferred onto the PVDF membranes (Bio-Rad). The membranes were incubated with primary antibodies for 2 hours, depending on the sensitivity of each antibody, washed with 1 × TBS at least 3 times, and then blotted with HRP-linked secondary antibody for 1 hour. The protein bands were visualized using a chemiluminescence detection system (Cell Signaling Inc.) In order to verify equal loading, PVDF membranes were stripped with Restore Western Blot Stripping Buffer (Themo fisher Scientific) and re-probed for the internal control protein beta-actin, which is constitutively expressed in both hepatoma cell types used in this study. The Integrated Density Value (IDV) of each band was quantitated using Bio-Rad ChemiDoc Image system (BioRad, Hercules, CA, USA).

### Statistical analysis

Each experiment was performed independently at least 3 times. The results are presented as the mean ± standard deviation (SD). The significance of the differences between the mean values was assessed by a two-tailed Student’s *t*-test using Microsoft Excel program or Prism GraphPad statistical analysis software (GraphPad, La Jolla, CA, USA). A *P*-value < 0.05 was considered statistically significant and is indicated by symbols in the figures and legends.

## SUPPLEMENTARY MATERIALS







## Abbreviations

Sf: Sorafenib
CA: carnosic acid
DCFH: 2′,7′-dichlorodihydrofluorescein
HCC: Hepatocellular carcinoma
ROS: reactive oxygen species
ERK1/2: extracellular signal-regulated protein kinase
LC3-II: LC3-phosphatidylethanolamine conjugate
PI: Propidium iodide
STAT: signal transducer and activator of transcription
CC: cell cycle
