# Supplementary material for: Carnosic acid increases sorafenib-induced inhibition of ERK1/2 and STAT3 signaling which contributes to reduced cell proliferation and survival of hepatocellular carcinoma cells
Source: Oncotarget. 2020 Aug 18;11(33):3129–43. doi: 10.18632/oncotarget.27687 (PMC7443370; doi:10.18632/oncotarget.27687)
Supplement: Supplementary file 1 [file oncotarget-11-3129-s001.pdf]

Carnosic acid increases sorafenib-induced inhibition of ERK1/2 and STAT3 signaling which contributes to reduced cell proliferation and survival of hepatocellular carcinoma cells

SUPPLEMENTARY MATERIALS

Supplementary Table 1: Effects of ERK1/2 and STAT3 inhibitors on the expression of regulatory proteins in Huh7 cells. See Supplementary Table 1

Supplementary Table 2: Effects of ERK1/2 and STAT3 inhibitors on the expression of regulatory proteins in HepG2 cells. See Supplementary Table 2
